# A rapid and sensitive recombinase aided amplification assay to detect hepatitis B virus without DNA extraction

**DOI:** 10.1186/s12879-019-3814-9

**Published:** 2019-03-05

**Authors:** Xin-xin Shen, Fang-zhou Qiu, Li-Ping Shen, Ten-fei Yan, Meng-chuan Zhao, Ju-Ju Qi, Chen Chen, Li Zhao, Le Wang, Zhi-shan Feng, Xue-jun Ma

**Affiliations:** 1 NHC Key Laboratory of Medical Virology and Viral Diseases, National Institute for Viral Disease Control and Prevention, Chinese Center for Disease Control and Prevention, Beijing, 102206 China; 2 Hebei Medical University, Shijiazhuang, 050031 Hebei China; 3 Children’s Hospital of Hebei Province, Shijiazhuang, 050031 Hebei China; 4 Myasthenia Gravis Research Institute, The First Hospital of Shijiazhuang, Shijiazhuang, 050011 Hebei China

**Keywords:** Hepatitis B virus, Detection, Recombinase aided amplification

## Abstract

**Background:**

Hepatitis B virus (HBV) infection is the major public health problem worldwide. In clinical practice, serological and molecular assays are the most commonly used diagnostic methods to detect HBV infection in clinical practices.

**Methods:**

Here we present a rapid and sensitive recombinase aided amplification assay (RAA) to detect HBV at 39.0 °C for 30 min without DNA extraction from serum samples. The analytical sensitivity of RAA assay was 100 copies per reaction and showed no cross reaction with human immunodeficiency virus (HIV) and hepatitis C virus (HCV). The universality of RAA assay was validated by testing of 41 archived serum samples with predefined HBV genotypes (B, C and D).

**Results:**

A total of 130 archived suspected HBV infected serum samples were detected by commercial qPCR with DNA extraction and RAA assay without DNA extraction (heat-treatment). Compared with qPCR assay as a reference, the RAA assay obtained 95.7% sensitivity and 100% specificity and a kappa value of 0.818.

**Conclusions:**

We developed a rapid, convenient, highly sensitive and specific method to detect HBV without DNA extraction in clinical samples. This RAA method of HBV detection is very suitable for clinical testing.

## Background

Hepatitis B virus (HBV) is an enveloped virus containing about 3.2 kb, partially double-stranded DNA genome and can be classified into eight genotypes (A to H) [1]. Different genotypes have distinct geographic distributions, genotype A is pandemic, B and C are mainly prevalent in Asia, D in southern Europe, E in Africa, F in the USA, and G in the USA and France. The newly discovered genotype H was found in central America [2]. Some studies indicated that different HBV genotypes revealed different disease profiles. Patients with genotype A had a higher rate of clearance HBV DNA and HBeAg than genotype D, and genotype A was more frequent with liver disease of death than genotype F [3]. Compared with genotype C, patients with genotype B had less active liver disease as HBV genotype B was more associated with earlier HBeAg seroconversion than genotype C [4].

Hepatitis B virus infection is a major public health problem worldwide, more than 240 million individuals are infected with chronic HBV, among untreated patients with chronic HBV infection, 15 to 40% may progress to cirrhosis [5]. As HBV is not eradicable by persons immune response or by antiviral drugs developed so far, the only preventive strategy is vaccination, however vaccine is unsuited with some patients, such as those with chronic kidney disease, human immunodeficiency virus infection, type I diabetes mellitus, and celiac disease [6] Therefore, development of potent HBV detection methods would provide a better insight into HBV immunopathogenesis and therapy [7] and guide clinical treatment and affect the prognosis [8]. To compared with other conventional molecular methods, the recombinase aided amplification (RAA) assay is a new isothermal amplification technology with the advantages of rapidity, simplicity and low-cost and therefore potentially suitable for clinical application. RAA has been successfully used to detect a variety of microbial pathogens [9, 10] and single nucleotide polymorphisms (SNP) [11]. In the RAA assay, there are there major proteins: single strand DNA binding protein (SSB), recombinase UvsX (*E. coli*) and DNA polymerase. The recombinase UvsX pairs the specific primers to template DNA, the main function of SSB is to protect the single chain template DNA and DNA polymerase is responsible for amplification and extension, and the amplification process is completed within 20–30 min at 39 °C.

In this study, we developed a RAA assay to detect HBV without DNA extraction. To our best knowledge, it is the first time to report RAA assay for detecting microbial pathogens without nucleic acid extraction.

## Methods

### Clinical samples

A total of 130 archived suspected HBV infected serum samples, 52 archived and typed HBV (13 genotypes B, 15 C and 13 D) positive serum samples [11], 10 archived serum samples from healthy donors, stocked human immunodeficiency virus (HIV, *n* = 5) RNA and hepatitis C virus (HCV, *n* = 6) RNA were obtained from Department of Viral Hepatitis of Chinese Center for Disease Control and Prevention. All the serum samples were collected between April 2016 to May 2017 and stored at − 80 °C. All the suspected HBV infected serum samples were HBsAg-positive and from chronic HBV patients (92 male, 38 female) aged from 28 to 46 years. This study was approved (#IVDC2016014) by the Institutional Review Boards of the National Institute for Viral Disease Control and Prevention, Center for Disease Control and Prevention of China.

### DNA extraction and heat treatment (without DNA extraction)

Total DNA was extracted from 200μL of each suspected HBV positive serum sample by using Master Pure Complete DNA and RNA purification kit (Epicenter Technologies, Madison, WI) according to the manufacturer’s instructions. The DNA samples were eluted in 50μL of nuclease-free water and stored at− 80 °C until use. In parallel, 1 μL of each serum sample was mixed with 9 μL of extraction buffer from the quick DNA extraction kit (Sangon Biotech, Shanghai, China), the 10μL of mixture was incubated at 80 °C for 3 min then cooled to room temperature until use.

### Primers and probes design

All the available HBV genomes were downloaded from GenBank databases. The sequences were aligned using Vector NTI 11.5.1. We designed HBV RAA primers and probes according to the principle of RAA primer and probe design. We used oligo7 software to carefully analyze dimer formation among themselves (self-dimers) and the formation of secondary structures (hairpins). Besides, we used Primer-BLAST and Blast of NCBI to check and ensure the specificity of primers and probe, respectively. Two optimal primers and one probe sequences are outlined in Table 1.

**Table 1:** The information on primer and probe of RAA assay

Primer/probe	Sequence (5′-3′)	Genomic position	Product size (bp)
F-primer	ATTCGCATGCCCCAACCTCAATCACTCACC	311–342	443
R-primer	AATACCACATCATCCATATAACTGAAAGCC	721–751	
Probe	TATCATCTTCCTCTTCATCCTGCTGCTATGCCTCA [FAM-DT][THF][BHQ-DT] TCTTGTT GGTTC [C3-spacer]	391–441	

Footnote; probe modifications: FAM, 6-carboxyfluorescein; THF, tetrahydrofuran; BHQ, black hole quencher; C3-spacer, 3′ phosphate blocker

### Construction of a recombinant plasmid

The targeted sequence (nt311-nt751, GenBank accession no. MH220971.1) was 440 base pair (bp) in length and cloning was done by TsingKe Biotech Corp (Beijing, China). The plasmid DNA was quantified using a NanoDrop (NanoPhotometer N60, Germany). The plasmids were serial 10-fold diluted ranging from 10^5^ to 10^1^ copy/μL and stored − 20 °C to evaluate the sensitivity of RAA assay.

### The RAA assay

The RAA amplification process was performed in 50 μL reaction volume using a commercial RAA kit (Qitian biological Co., Ltd., Jiangsu, China) which provides all the enzymes and reagents necessary for the amplification according to the manufacturer’s instruction. The reaction mixture contained 2 μL of extracted DNA template or 2 μL heat-treated mixture (without DNA extraction), 25 μL of reaction buffer, 15.7 μL of DNase-free water, 2.1 μL of primer F (10 μM), 2.1 μL of primer R (10 μM), 0.6 μL of probe (10 μM), and 2.5 μL of 280 mM magnesium acetate. The tubes were then transferred to the fluorescence detection equipment QT-F7200–0001 (Qitian, Jiangsu, China) set at 39.0 °C for 30 min. A positive control (HBV recombinant plasmid) and negative control (blank control) were included in each run.

### Analytical sensitivity, specificity, reproducibility and universality of RAA assay

The analytical sensitivity analysis of RAA assay was carried out using 10-fold serial dilutions of plasmid ranged from 10^5^ to 10^1^ copies/μL respectively. And the specificity was evaluated by testing of 11 HIV (*n* = 5), HCV RNA (*n* = 6) and 10 serum samples from healthy donors. Besides, twenty replicates were performed in different five days to validate the reproducibility of RAA assay. A total of 41 typed HBV samples containing genotypes B (*n* = 13), C (*n* = 15) and D (n = 13) were used to detect the universality of this method.

### Detection of clinical samples by RAA assay

A total of 130 suspected HBV infected serum samples were detected and quantified by a commercial qPCR kit (DAAN GENE, Guangzhou, China) according to the manufacture’s instruction. The same amounts of samples were also tested by regular RAA assay. The direct RAA assay was performed at the same time using heat-treated samples without DNA extraction.

## Results

### Analytical sensitivity and reproducibility of RAA assay

**Fig. 1:**
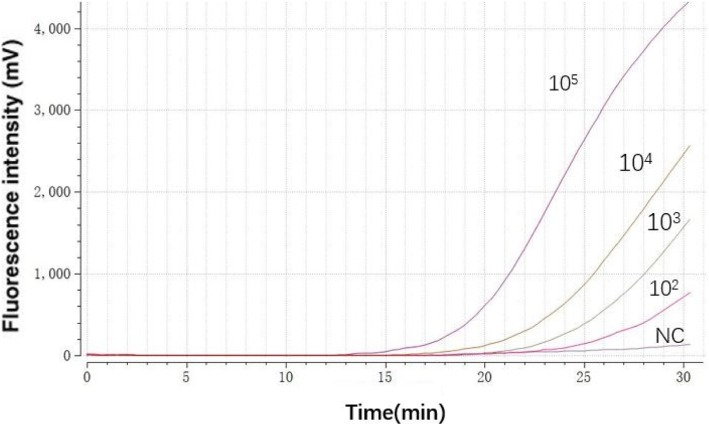
Representative HBV RAA reaction curve. A serial dilution ranging from 105 to 100 copies/reaction of recombinant plasmids. NC represents negative control

The analytical sensitivity of the RAA assay for HBV recombinant plasmids was approximately 10^2^ copies/reaction (Fig. 1). To validate the repeatability of RAA assay, 20 replicates of serially diluted HBV recombinant plasmids (10^5^–10^1^) were assayed and analyzed, 100 and 60% detection rates were achieved at concentrations from 10^5^ to 10^2^ copies/reaction and 10^1^ copies/reaction, respectively (Table 2).

**Table 2:** The reproducibility of RAA assay

Serial diluted HBV DNA	No. of replicates tested	No. of detection	Detection rate (%)
10^5^	20	20	100
10^4^	20	20	100
10^3^	20	20	100
10^2^	20	20	100
10^1^	20	12	60

### Analytical specificity and universality of RAA assay

The RAA assay for HBV was negative for HIV, HCV RNA and serum samples from healthy controls (data not shown) but positive for the 41 HBV infected serum samples previously typed by sequencing [11]. All of the samples (*n* = 13 genotype B, *n* = 15 genotype C, and n = 13 genotype D) can be detected by RAA assay regardless of extraction of nucleic acid or heat-treatment. Thus, the RAA assay demonstrated high specificity for the HBV and broad reaction to different HBV genotypes.

### Evaluation of RAA assay using serum samples

A total of 130 suspected HBV infected serum samples were tested with RAA assay by using extracted DNA and heat-treated samples. qPCR was also performed as a reference method using the same batch of samples. The qPCR tested 117 out of 130 samples positive with virus load range from 1.2 × 10^2^ IU/mL to 8.2 × 10^7^ IU/mL. The results of RAA assay with DNA extraction were in complete agreement with those obtained with qPCR. No false-positives were achieved between the RAA assay without DNA extraction and qPCR. Five samples with viral concentrations ranged from 1.2 × 10^2^ IU/mL to 4.8 × 10^2^ IU/mL mL were positive by qPCR but missed by RAA assay without extraction, displaying a slight decrease of sensitivity of direct RAA compared to qPCR. Compared with q-PCR, the sensitivity of RAA (heat-treatment) was 95.7% the 95% interval of confidence were 0.922–0.992 and the specificity was100%. The detailed comparison of detection results using three methods is present in Table 3.

**Table 3:** The detailed comparison of three methods by detecting clinical samples

Methods		q-PCR		Total	Kappa	*P*-value
		Positive	Negative			
RAA (heat-treatment)	Positive	112	0	112		
	Negative	5	13	18	0.818	0.025
	Total	17	13	130		
RAA (DNA extraction)	Positive	117	0	117		
	Negative	0	13	13	1.000	<0.001
	Total	117	13	130		

## Discussion

More than one-third of the world’s population has serological evidence of past or present HBV infection [12]. However, screening for HBsAg, anti-HBs and anti-HBc using enzyme-linked immunosorbent assay (ELISA) is not adequately effective to prevent transfusion-mediated HBV infections. What’s more, such method is normally both labor and time intensive. Nucleic acid tests (NATs) have now become a popular method to detect HBV in blood in the chronic phase of HBV infection including the window period as well as OBI [13]. Nested-PCR [14], real-time PCR [15], and loop-mediated isothermal amplification assay (LAMP) [16, 17] are commonly used NATs for HBV detection. Generally nested-PCR with tedious operation and easy to cross contamination is definitely not suitable for rapid clinical diagnosis. The real-time PCR is ideally used for monitoring the progression of disease and the effectiveness of treatment [2], but it’s difficult to be carried out in laboratories with poor financial condition, limited equipment and lack of skilled personnel.

LAMP assay shows better performance to detect HBV-DNA in resource-limited settings [16, 17]. For example, N.B.Quoc et al. [18] developed a closed tube loop-mediated isothermal amplification assay for rapid detection of hepatitis B virus in human blood at 65 °C in less than 60 min and each test costs 5.1$. In the present study, the RAA assay with DNA extraction revealed the ability to detect HBV infected clinical sample at 39 °C within 30 min. The agreement between qPCR assay and RAA assay with DNA extraction is 100%, and the cost is 3.7$ of each test. These results showed that the RAA assay was superior to LAMP assays for HBV DNA in terms of the rapidity, simplicity and cost. In addition, the universality of RAA assay in this study was extensively analyzed by using 41 pre-typed HBV samples, suggesting the RAA assay is well adaptable to detect HBV genotypes B, C, the most prevalent types in Asia.

The most attractive feature of proposed RAA assay in this study is to perform the RAA assay without DNA extraction. This avoided the traditional HBV-DNA extraction step and increased the likelihood of in field use of RAA assay. In our initial study, serum samples collected from HBV infected patients were heat-treated under different conditions using different extraction buffers from several commercial kits. We tested different combinations of several temperatures (75 °C, 80 °C and 85 °C,) and duration of time (1 min, 3 min and 5 min), The pretreatment condition was finalized at 80 °C for 3 min.in this study. Previous study reported on the LAMP detection of HBV [15] using heat-treated (up to 20 min) serum samples without universality analysis. In this study, we just needed less than 30 min to complete RAA detection using heat-treaded clinical samples (80 °C for 3 min) and the RAA sensitivity and specificity was 95.7 and 100% respectively, in comparison with qPCR, indicating that partly processed clinical samples can be used as target template for the RAA assay. Because no sophisticated equipment was required, this assay not only further reduced assay time and cost, but also simplified the detection process. It was even possible to perform the RAA assay combined with lateral flow dipstick (LFD) for visual detection in a closed fist using body heat without the need of any equipment and technical expertise [19].

## Conclusions

In conclusion, this study demonstrates that the RAA assay is a valuable alternative tool for detection of HBV DNA with the advantages of low cost, high sensitivity, easy implementation, and rapid detection. Given further clinical evaluation with larger sample size from different locations, the RAA assay without DNA extraction will be potentially adaptable for field detection and screening of HBV infection in clinical practice.

## Abbreviations

HBV: Hepatitis B virus
HCV: hepatitis C virus
HIV: human immunodeficiency virus
LFD: lateral flow dipstick
NATs: nucleic acid tests
RAA: recombinase aided amplification assay
SNP: single nucleotide polymorphisms
SSB: single strand DNA binding protein

## References

[1] Valsamakis A (2007). Molecular testing in the diagnosis and Management of Chronic Hepatitis B. Clin Microbiol Rev.

[2] Lai CL, Ratziu V, Yuen MF, Poynard T (2004). Viral Hepatitis.

[3] Sánchez-Tapias JM, Costa J, Mas A, Bruguera M, Rodés J (2002). Influence of hepatitis B virus genotype on the long-term outcome of chronic hepatitis B in western patients. Gastroenterology.

[4] CJ C, M H, AS L: Hepatitis B virus genotype B is associated with earlier HBeAg seroconversion compared with hepatitis B virus genotype C**.** Gastroenterology 2002, 122**:**1756–1762. 10.1053/gast.2002.33588 12055581

[5] Tang L, Covert E, Wilson E, Kottilil S (2018). Chronic hepatitis B infection: A review. Jama.

[6] Orlando R, Foggia M, Maraolo AE, Mascolo S, Palmiero G, Tambaro O, Tosone G (2015). Prevention of hepatitis B virus infection: from the past to the future. Eur J Clin Microbiol Infect Dis.

[7] Mohebbi A, Lorestani N, Tahamtan A, Kargar NL, Tabarraei A. An overview of hepatitis B virus surface antigen secretion inhibitors. Front Microbiol. 2018;9. 10.3389/fmicb.2018.00662 PMC5895781 29675010

[8] Liaw Y-F, Kao J-H, Piratvisuth T, Chan HLY, Chien R-N, Liu C-J, Gane E, Locarnini S, Lim S-G, Han K-H (2015). Erratum to: Asian-Pacific consensus statement on the management of chronic hepatitis B: a 2012 update. Liver Int.

[9] Chen C, Li XN, Li GX, Zhao L, Duan SX, Yan TF, Feng ZS, Ma XJ. Use of a rapid reverse-transcription recombinase aided amplification assay for respiratory syncytial virus detection. Diagn Microbiol Infect Dis. 2017;90. 10.1016/j.diagmicrobio.2017.10.005 29141771

[10] Zhang X, Guo L, Ma R, Cong L, Wu Z, Wei Y, Xue S, Zheng W, Tang S (2018). Rapid detection of Salmonella with recombinase aided amplification. J Microbiol Methods.

[11] Duan SX, Li GX, Li XN, Chen C, Yan TF, Qiu FZ, Zhao L, Zhao MC, Wang L, Feng ZS, Ma XJ. A probe directed recombinase amplification assay for detection of MTHFR A1298C polymorphism associated with congenital heart disease. Biotechniques. 64(5):211. 10.2144/btn-2018-2010 29793361

[12] Ocana S, Casas ML, Buhigas I, Lledo JL (2011). Diagnostic strategy for occult hepatitis B virus infection. World J Gastroenterol.

[13] Karimi G, Zadsar M, Vafaei N, Sharifi Z, Falahtafti M (2016). Prevalence of antibody to hepatitis B core antigen and hepatitis B virus DNA in HBsAg negative healthy blood donors. Virol J.

[14] Ambachew H, Zheng M, Pappoe F, Shen J, Xu Y (2018). Genotyping and sero-virological characterization of hepatitis B virus (HBV) in blood donors Southern Ethiopia. Plos One.

[15] Jiang W, Yue S, He S, Chen C, Liu S, Jiang H, Tong H, Liu X, Wang J, Zhang F (2018). New design of probe and central-homo primer pairs to improve TaqMan™ PCR accuracy for HBV detection.

[16] A A, SMR I, SU M. S T: detection of hepatitis B virus DNA among chronic and potential occult HBV patients in resource-limited settings by loop-mediated isothermal amplification assay. J Viral Hepat. 2018. 10.1111/jvh.12931 29768691

[17] Nyan DC, Ulitzky LE, Cehan N, Williamson P, Winkelman V, Rios M, Taylor DR (2014). Rapid detection of hepatitis B virus in blood plasma by a specific and sensitive loop-mediated isothermal amplification assay. Clin Infect Dis.

[18] Quoc, NB, Phuong, NDN, Chau, NNB, Linh, DTP. Closed tube loop-mediated isothermal amplification assay for rapid detection of hepatitis B virus in human blood. Heliyon 6;4(3). 10.1016/j.heliyon.2018.e00561 PMC5857714 29560471

[19] Liu L, Wang J, Geng Y, Wang J, Li R, Shi R, Yuan W. Equipment-free recombinase polymerase amplification assay using body heat for visual and rapid point-of-need detection of canine parvovirus 2. Mol Cell Probes. 2018. 10.1016/j.mcp.2018.04.004 PMC7127419 29705183

